# Changes in anterior femoral articular cartilage structure in collegiate rugby athletes with and without a history of traumatic knee joint injury following a five-month competitive season

**DOI:** 10.1038/s41598-021-94462-4

**Published:** 2021-07-26

**Authors:** Miyuki Hori, Masafumi Terada, Tadashi Suga, Tadao Isaka

**Affiliations:** 1 Graduate School of Sport and Health Science, Ritsumeikan University, 1-1-1 Noji-higashi, Kusatsu, Shiga 525-8577 Japan; 2 College of Sport and Health Science, Ritsumeikan University, 1-1-1 Noji-higashi, Kusatsu, Shiga 525-8577 Japan; 3 Research Organization of Science and Technology, Ritsumeikan University, 1-1-1 Noji-higashi, Kusatsu, Shiga 525-8577 Japan

**Keywords:** Biomarkers, Risk factors, Outcomes research, Musculoskeletal system

## Abstract

This study aimed to examine anterior femoral cartilage morphology before (pre-season) and after (post-season) a 5-month competitive season in collegiate ruby players with and without a previous history of traumatic injury to ligamentous, meniscus, and/or cartilage structures at the knee joint. Using a prospective cohort design, 42 male collegiate rugby players with a previous history of traumatic intracapsular knee joint injury and 124 players without knee injury history were included in this study. Ultrasonography assessments of anterior femoral cartilage were performed before (pre-season) and following a 5-month athletic season (post-season). Rugby players with a history of traumatic knee joint injury had greater lateral condylar thickness (2.37 ± 0.35 mm, *p* = 0.03), intercondylar thickness (2.51 ± 0.47 mm, *p* = 0.03), and partial area (44.67 ± 7.28mm^2^, *p* = 0.02) compared to control players (lateral = 2.23 ± 0.35 mm, intercondylar = 2.32 ± 0.47 mm, partial area = 41.60 ± 7.26 mm^2^), regardless of pre-and post-season assessment time points. Pre-season ultrasonography assessment of lateral condylar thickness (2.34 ± 0.47 mm, *p* = 0.02), medial condylar thickness (2.05 ± 0.43 mm, *p* = 0.03), and partial area (44.10 ± 9.23 mm^2^, *p* = 0.001) were significantly greater than the post-season ultrasonography assessment time point (lateral = 2.26 ± 0.43 mm, medial = 1.98 ± 0.43 mm, partial area = 42.17 ± 8.82 mm^2^), regardless of group membership. Rugby players with a history of intracapsular knee joint injury displayed altered anterior femoral cartilage size via ultrasonography assessments. Regardless of a presence of injury history, collegiate rugby players showed a decrease in cartilage thickness and partial area following a 5-month competitive season.

## Introduction

Participation in regular physical activity and organized team sports has an important role in maintaining articular cartilage health and slowing the progression of lower extremity joint osteoarthritis (OA)^1,2^. However, participation in certain sports may increase the risk of OA^3–5^. A high prevalence of knee OA has been reported in contact and collision sports such as soccer^3–5^ and rugby^6^. Given the intensity of physical contact and collision sports, the incidence of a traumatic knee joint injury is high in rugby^7–9^ and may lead to long-term morbidity^6,10^, subsequently increasing the number of years living with disability. The strong association of traumatic knee intracapsular injury with symptomatic radiographic OA has been observed in rugby^6^, indicating that rugby-related injury is one of the risk factors associated with the development of early onset knee OA. Therefore, understanding of the mechanisms accelerating the development of OA following traumatic knee intracapsular injury is important for effective administration of effective prevention for post-traumatic knee OA in rugby cohorts.

While the development of early onset knee OA following traumatic joint injury is a multifactorial progressive process, researchers have theorized that alterations in a cartilage response to mechanical loading may accelerate the development of knee OA. Alterations in lower extremity biomechanics resulting from knee injury may alter cartilage response to mechanical joint loads that occurs during activities of daily living and sport-related activities^11–16^, which adversely affects tissue homeostasis and structural integrity of cartilage^17–23^. Investigators have observed increased strain of the tibiofemoral cartilage compartment during a weight-bearing activity in individuals with medial meniscus injury^24^. Contact and collision sports with high prevalence of knee OA involves a greater frequency of abrupt cutting, decelerating, and accelerating movements that exerts greater aberrant shear or torsional loading, which is theorized to contribute to reducing the fatigue life of cartilage^25^. Thus, it is critical to understand how cartilage of rugby players with knee injury responds to rugby-related activities.

Quantifying the ability of the cartilage structure to appropriately respond to mechanical loading has been considered as a sensitive marker to detect the earliest alterations in cartilage structure and function^26–28^. Ultrasonography (US) has been utilized as a valid and reliability imaging tool to assess the femoral cartilage size and the amount of cartilage deformation after physical activity^26,27,29,30^. Previous investigations have demonstrated high agreement between a US method and cross-sectional cadaver measurements^29^ as well as magnetic resonance imaging (MRI)^30,31^. Harkey et al.^32^, using US, observed increased thickness of anterior femoral cartilage in individuals who have received surgical management of anterior cruciate ligament (ACL) tear compared with uninjured control individuals. Previous studies reported that acute deformation of the anterior femoral cartilage was greater after common physical activity conditions compared to non-loading conditions^26,27^. One cohort study assessed a longitudinal change in femoral cartilage thickness using MRI in physically active young adults with ACL injury and observed an increase in cartilage thickness over 5 years following ACL injury^33^. However, no investigators to data have used US to assess the short-term longitudinal cartilage response to physical activity in rugby players who have previously experienced traumatic knee joint injury. Understanding associations between knee injury and the longitudinal response of the femoral cartilage to sport-related activities will direct clinicians and researchers to determine optimal exercise-related strategies to mediate the risk of post-traumatic knee OA. Therefore, the purpose of this study was to investigate the effects of the combination of a previous history of traumatic knee joint and competitive athletic activities on the anterior femoral cartilage morphology in collegiate rugby players. Based on previous investigations^32,33^, we hypothesized that collegiate rugby players with a history of traumatic knee joint injury would demonstrate a greater increase in anterior femoral cartilage thickness and partial area following a competitive season compared with control players.

## Results

All included participants completed the follow-up, post-season assessment. Anthropometric characteristics were not different between the knee joint injury history and control groups (*p* > 0.05, Table 1). Participants with a history of knee joint injury scored significantly lower on all subscales of the Knee Osteoarthritis Outcome Scores (KOOS) compared to controls (*p* < 0.05, Table 1).

Means and standard deviations of US anterior femoral cartilage variables at pre-and post-season measurements can be found in Table 2. There were significant group main effects for lateral condylar thickness (F_1,164_ = 4.62; *p* = 0.03; β = 0.57), intercondylar thickness (F_1,164_ = 5.14; *p* = 0.03; β = 0.62), and partial area (F_1,164_ = 5.59; *p* = 0.02; β = 0.65). Rugby players with a history of traumatic knee joint injury had greater lateral condylar (2.37 ± 0.35 mm) and intercondylar thickness (2.51 ± 0.47 mm), as well as greater partial area (44.67 ± 7.28mm^2^) compared to control players (lateral = 2.23 ± 0.35 mm, intercondylar = 2.32 ± 0.47 mm, partial area = 41.60 ± 7.26 mm^2^), regardless of pre-and post-season assessment time points. The magnitude of between-group differences was moderate for lateral condylar thickness (*g* = 0.40; 95% confidence intervals (CIs) = 0.05, 0.75), intercondylar thickness (*g* = 0.40; 95% CIs = 0.05, 0.75), and partial area (*g* = 0.42; 95% CIs = 0.07, 0.77). No significant group main effects were not observed for medial condylar thickness (F_1,164_ = 2.86; *p* = 0.09; β = 0.39; *g* = 0.21; 95% CIs =  − 0.14, 0.56, knee injury = 2.07 ± 0.20 mm, control = 1.96 ± 0.59 mm) or echo intensity (F_1,164_ = 0.37; *p* = 0.54; β = 0.09; *g* = – − 0.11; 95% CIs =  − 0.46, 0.24, knee injury = 30.83 ± 10.32, control = 31.95 ± 10.37) with small effect sizes.

**Table 1:** Demographic characteristics for the traumatic knee joint injury history and control groups, mean (standard deviation).

	Knee joint intracapsular injury history	Control	*p* value
n	42 males	124 males	–
Age (year)	19.98 (1.22)	19.76 (1.15)	*p* = 0.30
Height (cm)	173.26 (6.04)	174.40 (6.28)	*p* = 0.31
Body mass (kg)	84.64 (12.42)	84.12 (11.91)	*p* = 0.81
Body mass index (kg/m^2^)	28.15 (3.65)	27.60 (3.22)	*p* = 0.36
Body fat percentage (%)	23.75 (17.17)	21.24 (4.37)	*p* = 0.14
# of previous knee injuries	1.67 (0.82)	0.00	–
	Min = 1, Max = 4		
Time since the most recent knee joint injury (month)	19.94 (16.09)	–	–
**Knee Injury and Osteoarthritis Outcome Score**			
Pain	96.15 (5.15)	98.44 (4.33)	*p* = 0.03*
Symptoms/Stiffness	89.18 (11.98)	95.60 (7.84)	*p* < 0.01*
Function, Activities of Daily Living	99.16 (2.39)	99.84 (0.66)	*p* = 0.08
Function, Sports, and Recreational Activities	89.40 (18.19)	98.64 (4.51)	*p* < 0.01*
Quality of Life	90.03 (16.68)	97.94 (8.25)	*p* < 0.01*

*The knee injury history group exhibited less scores on the Knee Injury and Osteoarthritis Outcome Score compared with the control groups (*p* < 0.05).

**Table 2:** Femoral cartilage variables at pre-and post-season assessments for the knee injury history and control groups.

Mean (standard deviation)	Pre-season					Post-season				
	Thickness			Partial Area	Echo Intensity	Thickness			Partial Area	Echo Intensity
	Lateral	Intercondylar	Medial			Lateral	Intercondylar	Medial		
Knee Injury	2.38*^#^	2.51*	2.10^#^	45.44*^#^	30.33	2.35*^#^	2.52*	2.04^#^)	43.90*^#^	31.32
(n = 26)	(0.48)	(0.54)	(0.43)	(8.90)	(14.23)	(0.45)	(0.55)	(0.41	(8.65)	(13.03)
Control	2.30*^#^	2.40*	2.00^#^	42.76*^#^	33.30	2.16*^#^	2.24*	1.93^#^	40.44*^#^	30.60
(n =124 )	(0.40)	(0.54)	(0.37)	(7.72)	(13.90)	(0.36)	(0.52)	(0.37)	(7.33)	(9.93)

*Significant main effects for group (*p* < 0.05) indicate that the knee injury group had greater lateral and intercondylar thickness as well as partial area than the control group, regardless of pre-and post-season time points. ^#^Significant main effects for time (*p* < 0.05) indicate that pre-season femoral cartilage values were greater than post-season values, regardless of group.

The following pre-season US femoral cartilage variables were significantly greater than post-season variables, regardless of group membership; lateral condylar thickness (F_1,164_ = 5.54; *p* = 0.02; β = 0.65; *g* = 0.18; 95% CIs =  − 0.04, 0.39, pre = 2.34 ± 0.47 mm, post = 2.26 ± 0.43 mm), medial thickness (F_1,164_ = 5.03; *p* = 0.03; β = 0.61; *g* = 0.16; 95% CIs =  − 0.05, 0.38, pre = 2.05 ± 0.43 mm, post = 1.98 ± 0.43 mm), and partial area (F_1,164_ = 11.51; *p* = 0.001; β = 0.92; *g* = 0.21, 95% CIs = 0.00, 0.43, pre = 44.10 ± 9.23 mm^2^, post = 42.17 ± 8.82mm^2^).

There were no significant time main effects for intercondylar thickness (F_1,164_ = 2.57; *p* = 0.11; β = 0.36; *g* = 0.12; 95% CIs =  − 0.10, 0.33, pre = 2.45 ± 0.61 mm, post = 2.38 ± 0.60 mm) or echo intensity (F_1,164_ = 0.43; *p* = 0.51; β = 0.10; *g* = 0.06; 95% CIs =  − 0.16, 0.27, pre = 31.82 ± 16.10, post = 30.96 ± 12.42). There were no significant group × condition interactions for lateral condylar thickness (F_1,164_ = 1.92; *p* = 0.17; β = 0.28), medial thickness (F_1,164_ = 0.07; *p* = 0.79; β = 0.06), intercondylar thickness (F_1,164_ = 3.28; *p* = 0.07; β = 0.44), partial area (F_1,164_ = 0.46; *p* = 0.50; β = 0.10), or echo intensity (F_1,164_ = 2.00; *p* = 0.16; β = 0.29).

## Discussion

Participation in sports and regular physical activity does not necessarily increase a risk of knee OA^25^, but traumatic knee joint injury sustained during athletic activity has been recognized as a risk factor for knee OA^6,34^. The present study aimed to examine the short-term longitudinal changes in US femoral cartilage variables in an athletic population with and without a previous history of traumatic knee joint injury. Our main findings were that anterior femoral cartilage thickness was associated with a previous history of knee intracapsular injury in an athletic population. Specifically, collegiate rugby players with a previous history of knee intracapsular injury demonstrated greater lateral condylar and intercondylar thickness, as well as partial area, compared to control players, regardless of pre-and post-season assessment time points. The moderate effect size values for lateral thickness, medial thickness, and partial area, with CIs that did not cross zero, suggests clinically meaningful differences between groups may be present. A previous investigation has reported greater medial and lateral condylar thickness and greater cross-sectional area in individuals with a history of ACL reconstruction compared to control participants^32^. Furthermore, previous authors demonstrated longitudinal increases in medial femoral cartilage thickness at 1 year^35^, 2 years^36^, and 5 years following ACL injury^33^. Consistent with the findings of previous investigations of ACL injury populations, our findings from the current study indicates that traumatic knee intracapsular injury may alter the macrostructure of the femoral cartilage in rugby players. This study provides evidence that supports the need for future investigations to longitudinally monitor cartilage structure when determining the association between joint health and traumatic knee joint injury in competitive athletic populations.

We observed that both the knee injury history group and control groups exhibited decreased femoral cartilage thickness and partial area following a 5-month competitive season. This finding suggests monitoring changes in femoral cartilage macrostructure during an athletic season may be a critical step towards the creation of optimal exercise-related strategies to mediate the risk of knee OA. While the smalleffect sizes, with CIs that crossed zero, suggest differences in cartilage thickness between pre-and post-season may be not clinically meaningful, our findings of the amount of changes in femoral cartilage thickness on US are somewhat similar to those of a previous study in which authors demonstrated that femoral articular cartilage had longitudinal change their structures in mature volleyball athletes^37^. Specifically, Eckstein et al.^37^, observed mature volleyball athletes displayed a decrease in medial (− 0.32 mm) and lateral cartilage thickness (− 0.16 mm) at 2-year follow-up assessments. These findings from the previous^37^ and current studies may indicate that the effects of physical activity may lead to pronounced short-term and long-term differences in macrostructure of femoral cartilage. Participation in competitive sport might be associated with the greater frequency of shear or torsional loading induced by abrupt lateral, decelerated, and accelerated movements^6^. The shear or torsional load accumulated by sport-related activities for a longer period may cause chronic changes in the femoral cartilage. This is on speculative and future studies are needed to examine the long-term effect of various modes of physical activities and different knee joint loading on femoral cartilage macrostructure.

The unique aspect to this study is that we assessed a short-term longitudinal change in the anterior femoral cartilage size following a 5-month competitive season in collegiate rugby players with a history of knee injury. However, no significant interactions between Injury and Time were observed for all femoral cartilage outcome measures. These findings indicate that players with a knee joint injury history may not present with a progressive thickening or thinning of anterior femoral cartilage during a competitive season. We speculate that no progressive changes in anterior femoral cartilage in rugby players with knee injury history might be due to movement strategies they utilized as an effort to protect the knee joint. Rugby athletes in the knee injury history group received supervised physical rehabilitation following a knee injury. It is possible that those with a history of knee injury have restored proper knee function and the ability to attenuate the external loads following supervised rehabilitation programs. Furthermore, previous researchers have suggested that individuals, who have received surgical management following ACL injury, may offload the injured limb to avoid placing excessive stress on injured joint in order to minimizing a risk for further tissue damage or re-injury^38,39^. This compensation mechanism may influence mechanical joint loading of the cartilage and its structure in the injured knee. The explanations are speculative. Clearly, a prospective investigation is needed to determine if movement strategies and joint loading patterns in rugby athletes with a knee injury history are associated with femoral cartilage thickness and cross-section area.

While we assessed the short-term cartilage response immediately after a competitive season, we are unaware of the degree to which femoral cartilage could recover its macrostructure following a period of lower loading intensity and duration, such as off-season. A previous investigation observed slower recovery to baseline cartilage morphology following cartilage deformation induced by higher intense activity^27^. The high resilience of cartilage can minimize the risk of cartilage failure^40^, and slower recovery of femoral cartilage structure after removing loading accumulated by physical activity is one of factors that increase a risk for knee OA^14^. Cartilage recovery following the season could differ between rugby players with and without a history of traumatic knee joint injury. Additional assessment of cartilage recovery after the athletic season (i.e., assessing cartilage macrostructure following off-season) as an indicator of cartilage resiliency may better monitor longitudinal cartilage conditioning of rugby players with a knee joint injury history following a long period of high intense activity. Future studies should examine both cartilage response and recovery following the repetitive loading induced by cutting, deceleration, and acceleration movements in athletic populations with traumatic knee joint injury.

While we observed a significant decrease in lateral and medial condyle thickness and cross-sectional area following a 5-month competitive season, these structural changes were not accompanied by alterations in echo intensity. Echo intensity may be sensitive to change in the hydration status of cartilage and cartilage loss is associated with fluid exudation^41^. Previous study reported echo intensity measures were associated with a presence of arthroscopic-based cartilage damage^42^. Future investigations are needed to validate US echo intensity as a clinically accessible assessment tool to detect longitudinal changes in cartilage water content and cartilage composition and monitor joint tissue health.

In the current study, there are some limitations and caveats that open the door for future research. The heterogeneity of severity in intracapsular injury, the time since the most recent injury, and injury types makes it difficult to generalize findings to one specific type of knee joint intracapsular injury, as these are factors that may influence the results of our study. The average time since knee intracapsular injury in our participants was 19.94 ± 16.09 months, and the range of time since knee intracapsular injury was wide (2–59 months). Therefore, we ran a post-hoc exploratory Pearson product moment correlation analyses to evaluate the association between time since intracapsular injury and anterior femoral cartilage morphology using data from the current study. We found negligible negative correlations of the anterior femoral cartilage thickness and partial area with time since injury in players with a history of intracapsular injury (r =  − 0.03 ~ 0.11, *p* > 0.05). However, future studies are required to examine interaction between injury and competitive athletic activities for anterior femoral cartilage macrostructure in a more homogenous injury cohort.

While all players participated in the same practice, conditioning, and training sessions, we did not consider other possible influential factors such as game hours played, position played, and other physical activities in which players participated beside rugby team activities. While there were no differences in game and practice days participated between players with and without a history of knee injury (*p* > 0.05), the cumulative load during this investigation may be different among participants. Furthermore, we did not assess knee alignment (e., knee valgus) that influences the femoral cartilage thickness and contributes to the progression of knee OA^43^. Future investigations will need to include the factors identified above to comprehensively understand the effects of knee joint intracapsular injury on morphological characteristics of anterior femoral cartilage in this athletic population. Observer bias may be introduced into the US image analysis by an unblinded examiner performing all of the US image analyses. Future studies should conduct with a blinded design as examiners are blinded to group memberships. Lastly, an US does not allow for capturing the enter cartilage at the tibiofemoral and patellofemoral joint, and we could determine effects of knee joint injury and competitive season on thickness and size in other femoral cartilage compartments. While an US method utilized by this study has been accepted for viewing the lateral and medial femoral condyle as well as intercondylar notch^29–31^, anatomically this method is limited to capture the most anterior position of the femoral cartilage, which may represent both patellofemoral and tibiofemoral articulations.

## Conclusion

We observed that collegiate rugby athletes with a previous history of knee intracapsular injury had greater anterior femoral cartilage lateral condyle thickness, intercondylar thickness, and partial area compared with uninjured control players. Traumatic intracapsular injury at the knee joint may alter the macrostructure of the anterior femoral cartilage in rugby players, which may affect the long-term joint and cartilage health. Short-term longitudinal changes in anterior femoral cartilage structure were observed in collegiate rugby athletes following a competitive season, regardless of the presence of a knee injury history. These findings suggest regular femoral cartilage health assessments during an athletic season may be a critical step towards the creation of optimal exercise-related strategies to mediate the risk of knee OA.

## Materials and methods

### Study design

**Figure 1:**
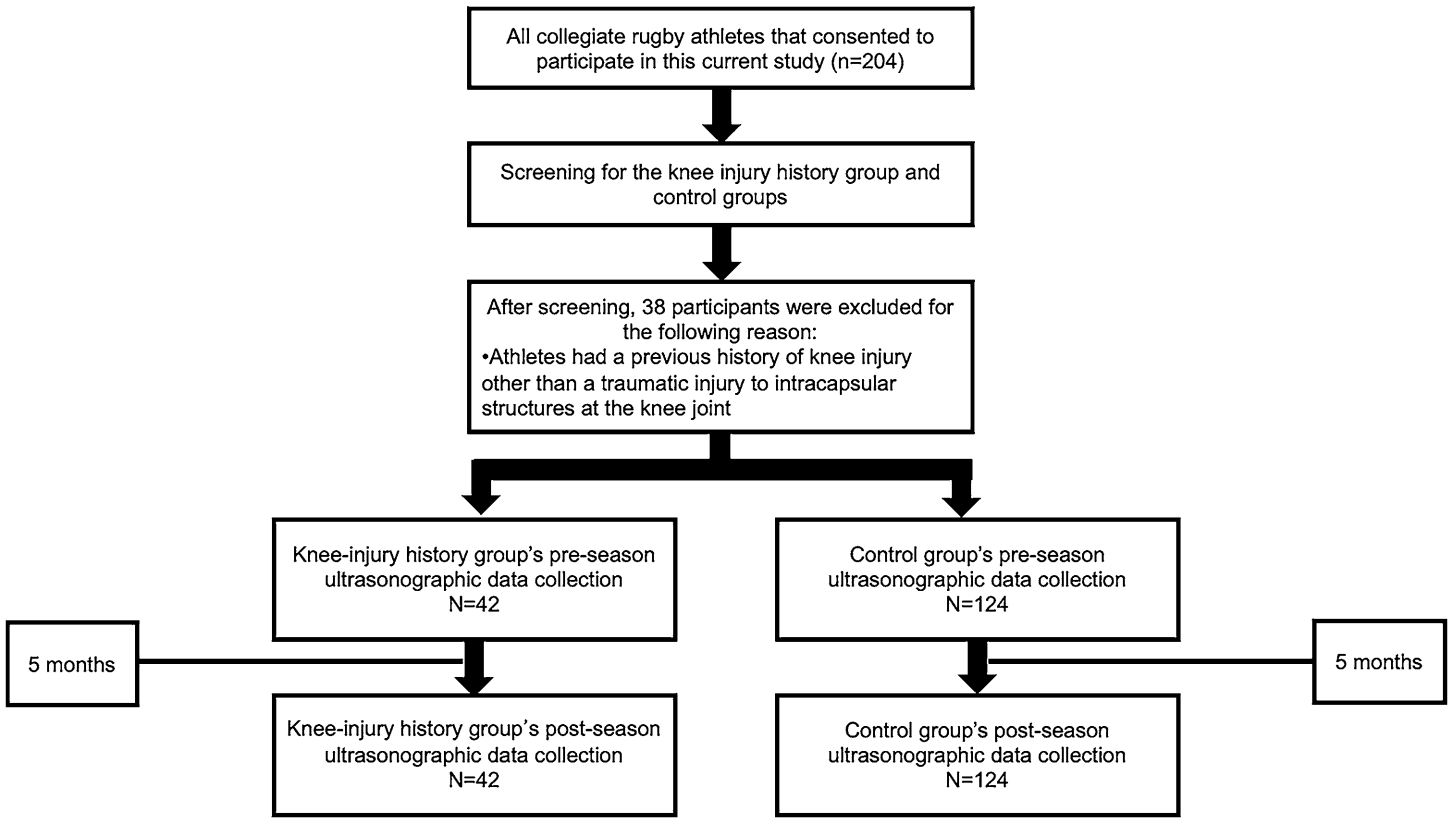
Flow chart depicting rugby player participation, data loss, and time between femoral cartilage ultrasonography outcome measures data collection sessions.

A prospective observational design was used to examine US outcome measures of anterior femoral cartilage morphology before (pre-season) and after (post-season) a 5-month competitive season (108 days of practice and 17 games) in collegiate ruby players with and without a previous history of knee joint injury (Fig. 1). A single, unblinded investigator performed all femoral cartilage US assessments. The methods of this study were carried out in accordance with the Declaration of Helsinki. All methodological protocols were approved by the Ethical Committee on Human Research of the Ritsumeikan University Institutional Review Board.

### Participants

Two hundred four rugby players from a Division I collegiate team were enrolled in this study. All players were cleared for full participation by a physician. A written informed consent was obtained from all participants, along with parental or guardian consent if necessary. Enrolled players were screened with self-reported questionnaires and a medical chart recorded by an athletic trainer providing care to the rugby team to allocate the knee injury history group or control group. Rugby players in the knee injury history group were required to have a previous history of at least one significant traumatic injury to intracapsular structures (e.g., anterior and posterior cruciate ligament sprain, lateral and medial meniscus injury, femoral cartilage injury) in the knee joint resulting in swelling, pain, and temporal loss of function. No participants in the knee injury history group had acutely injured their knee in the previous 1 month of pre-season testing. As this study was focused on effects of a history of traumatic intracapsular injury at the knee joint, rugby players that have a previous history of extracapsular knee injuries (e.g., medial and lateral collateral ligament sprains) other than an intracapsular injury at the knee joint (n = 38) were not included in this current study. The type and frequency of injuries sustained by the participants are reported in Table 3. The control group participants were required to have no previous history of: 1) musculoskeletal injuries in the lower extremity; 2) back pain; and 3) surgery in the lower extremity. This resulted in a total of 166 rugby players, 42 in the knee injury history group and 124 in the control group (Table 1).

**Table 3:** List of a previous history of a traumatic knee joint injury.

Injury type	Frequency	The number of surgical management
Anterior cruciate ligament (ACL) sprain	19	13
Posterior cruciate ligament (PCL) sprain	13	0
Medial meniscus injury (MMI)	12	10
Lateral meniscus injury (LMI)	9	7
Femoral cartilage injury	1	1

The number of participants who sustained multiple knee joint injury:

ACL + MMI = 4, ACL + LMI = 2, MMI + LMI = 4, LMI + cartilage = 1.

Additional information related to each participants’ perception of their knee were collected for both groups using the KOOS. The KOOS is a valid and reliable region-specific patient-generated instrument and consists of 42-items across 5 subscales that assess symptoms , pain, activities of daily living , sport and recreation, and quality of life ^44,45^.

### Data collection procedures

#### Ultrasonographic image acquisition

**Figure 2:**
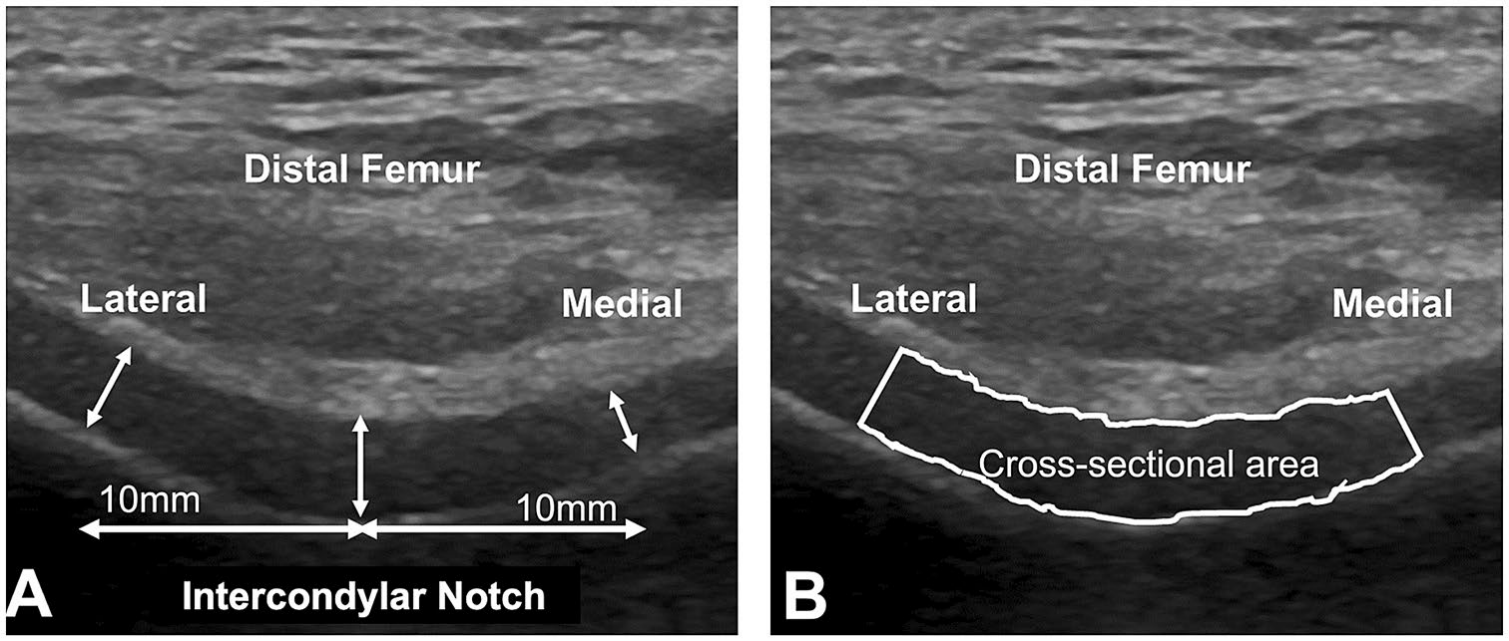
Ultrasonographic image of the anterior femoral articular cartilage: (**A**) Articular cartilage thickness. (**B**) Articular cartilage partial area.

Participants laid supine comfortably on an examination table with the testing knee positioned in the 140° flexion, controlled using a handheld goniometer^26^. A portable US unit (LOGIQe V2; General Electric Co., Fairfield, CT, USA) with a 12-MHz linear probe was used to obtain US images of the anterior femoral cartilage for this study. All system settings of the portable US unit were consistent between pre-season and post-season testing sessions as well as participants. The liner probe was placed anteriorly over the distal femoral cartilage of the medial and lateral femoral condyles in the axial position and above the superior margin of the patella and oriented to obtain the maximum reflection of the femoral trochlea and the overlying hyaline cartilage as a previous study reported^26,27,29,46^. The location at which the intercondylar notch was centered on the screen was marked on the skin, enabling the examiner to return the probe to the exact location for all subsequent scans. Three images were recorded at each testing session (pre-and post-season), with the linear probe being removed and repositioned on the knee between recorded images (Figure 2). The US examination of the distal femoral cartilage variables has been demonstrated to be valid^29–31^ and reliable (ICC=0.83–0.99)^27^. Further, prior to data collection for the current study, we conducted a pilot study to establish a priori intratester reliability that included 6 participants and used the US image acquisition and processing as described in this study. We performed three US examination sessions separated by at least 1 week, and an US examiner (M.H.) established good to excellent intratester reliability (ICC_2,3_=0.82–0.96).

#### Ultrasonographic images processing

A single investigator (M.H.) assessed distal femoral cartilage thickness, partial area, and echo intensity using ImageJ software (National Institutes of Health, Bethesda, MD, USA).

##### Anterior distal femoral cartilage thickness

Anterior distal femoral cartilage thickness was measured at the intercondylar notch and 1 cm apart in the medial and lateral directions that were used as an estimate of the medial and lateral condyle cartilage thickness^47^. The straight-line distance (mm) drawn from the hyperechoic cartilage–bone interface to the synovial space–cartilage interface was used to measure femoral cartilage thickness (Fig. 2A)^26,27^.

#### Partial area and echo intensity

Partial area (mm^2^) of the femoral cartilage was assessed by manually tracing an area of femoral cartilage between a lateral and a medial measurement point where femoral cartilage thickness was measured (Fig. 2B). Echo intensity was determined by the average gray-scale value across all pixels in the selected area on scale from 0 to 255^48,49^.

### Statistical analysis

An a *priori* alpha level was set at *p* < 0.05 using SPSS 26.0 (SPSS, Inc. Chicago, IL.) for all statistical tests. Anthropometric variables and the KOOS scores were compared between the knee joint history and control groups using independent t-tests. Separate 2 × 2 (group × time) repeated measure ANOVAs were used to analyze each US femoral cartilage variable. In the case of statistically significant interactions, a post hoc univariate analysis with pairwise comparison was conducted to ascertain the location of significant differences. For the knee joint injury history group, the previously injured limb was used. For the control group, no side-to-side differences existed for any of the US femoral cartilage measures (*p* > 0.05); therefore, the mean of both sides from the players without a history of knee joint were used. To assess the magnitude of differences in each US femoral cartilage measure between independent variables, Hedges’ *g* using the pooled standard deviations were calculated, along with 95% confidence intervals for each pairwise comparison. The strength of the effect sizes was interpreted as small (0 ≤ *g* < 0.40), moderate (0.40 ≤ *g* < 0.70), large (*g* ≥ 0.70) with 95% CIs^50^.
